# Sequential haematological and serum biochemical changes in Black Bengal goats infected with a local isolate of *peste des petits ruminants* virus from Bangladesh

**DOI:** 10.1002/vms3.373

**Published:** 2020-10-03

**Authors:** Shahana Begum, Mohammed Nooruzzaman, Azmary Hasnat, Mohammad Rafiqul Islam, Emdadul Haque Chowdhury

**Affiliations:** ^1^ Department of Pathology Faculty of Veterinary Science Bangladesh Agricultural University Mymensingh Bangladesh

**Keywords:** Black Bengal goats, electrolyte imbalance, enzymes, experimental infection, haematology, PPR

## Abstract

**Background:**

Knowledge of sequential changes in haematobiochemical parameters of infected animals helps in the formulation of appropriate supportive therapy.

**Objective:**

We investigated the sequential haematological and biochemical changes in peste des petits ruminants (PPR)‐infected Black Bengal goats.

**Methods:**

Goats were either infected with PPR virus (PPRV; *n* = 8) or sham infected with sterile phosphate‐buffered saline (*n* = 4) via the intranasal route. Blood and sera were collected from both groups at different days post‐infection (dpi) and analysed. Goats were sacrificed at different dpi and the amount of PPRV RNA in different tissues was quantified by real‐time RT‐PCR.

**Results:**

The PPRV‐infected goats showed mild depression and scanty nasal secretions starting at 4 dpi which became severe with high fever (106°F), dyspnoea, stomatitis, profuse orinasal discharge and diarrhoea at 9–13 dpi. PPRV RNA was detected in different tissues of infected goats. Severe lymphocytic leukopenia (at 18 dpi) was observed in infected goats. Total protein and albumin decreased in infected goats starting at 10 dpi. An elevated level of enzymes (alkaline phosphatase, creatine kinase, aspartate transaminase and alanine transaminase) and metabolites (blood urea nitrogen and urea B) were found in infected goats starting at 7–10 dpi, suggesting damages in the liver and kidneys. PPR‐infected goats showed elevated sodium and chloride ions starting at 7 dpi. The majority of infected goats were seroconverted by 14 dpi.

**Conclusions:**

Anti‐diarrheal agents, aqua solutions and other medicine to support liver and kidney functions could be considered as supportive therapy against PPRV infection.

## Graphical abstract

The sequential haematobiochemical changes in a Bangladeshi PPR virus strain was studied in Black Bengal goats upon experimental infection. The PPRV RNA was detected in different tissues of the infected goats. Infected goats showed high fever, nasal discharges, stomatitis and diarrhoea at 9–13 dpi. Leukopenia, hypoproteinemia, elevated serum enzymes and metabolites and electrolyte imbalance were found in the infected goats. The findings would guide in preparing appropriate supportive therapy against PPRV infection.

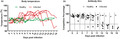

## 1 INTRODUCTION

In Bangladesh, goat rearing has been practiced in both rural and urban areas for decades and the goat is considered as poor man's cow because of its immense contribution to the livelihood of rural people (Ahmed, 2017). The goat offers a supply of quality meat, milk and hide that contribute to the national economy. In the year 2017–2018, there were about 26.1 million goats in Bangladesh of which about 90% were Black Bengal goats (BBS, 2019). Extreme climatic conditions such as heavy rainfall and flood, lack of grazing lands, parasitic infestation and outbreaks of various infectious diseases hinder successful goat farming in Bangladesh (Ahmed, 2017). Peste des petits ruminants (PPR), the number one killer of goats, is considered as one of the major constraints in sustainable goat farming in Bangladesh (Siddiky, 2013). The first outbreak of PPR in Bangladesh was reported in 1993 (Islam et al., 2001). Since then, outbreaks of PPR have regularly been reported across the country (Begum et al., 2018; Bhuiyan et al., 2014; Chowdhury et al., 2014; Rahman et al., 2011, 2016). Provision of inadequate veterinary services combined with poor diagnostic facilities stalled the successful PPR control programme (Haider et al., 2017).

The prognosis of acute PPR is usually very poor, especially when aggravated with secondary bacterial infection, poor nutrition and other stresses (Saliki, 1988). Supportive therapy with fluid, electrolytes and antibiotics to prevent secondary bacterial infection can reduce the mortality of PPR (Chowdhury et al., 2014; Yousuf et al., 2015). Knowledge of changes in the haematobiochemical parameters of PPR‐infected goats is necessary to formulate appropriate supportive treatment (Das et al., 2015). In addition, based on haematobiochemical changes at different stages of the disease, a different treatment plan will be necessary.

Several studies have been conducted to assess haematobiochemical alterations of goats died of natural PPR outbreaks and generated conflicting findings (Das et al., 2015; Sharma et al., 2012). The progressive changes in the haematobiochemical parameters of goats infected with PPR could not be evaluated on natural PPR infection. Therefore, the study was designed to assess the sequential haematological and serum biochemical alterations of Black Bengal goats experimentally infected with a Bangladeshi strain of PPRV.

## 2 MATERIALS AND METHODS

### 2.1 Animals

Twelve healthy Black Bengal goats aged between 4 and 8 months with a history of no vaccination against PPR were purchased from local markets. Animals were kept in relative isolation for 2 weeks with adequate feed and water. The goats were dewormed using subcutaneous ‘Ivermectin’ injection as per manufacturer's recommendation. All goats were tested for anti‐PPRV antibodies with a commercial competitive ELISA kit (ID Screen PPR Competition, ID‐Vet, Montpellier, France) and found seronegative for PPRV.

### 2.2 Ethics statement

All applicable national and institutional guidelines for the care and use of animals were followed. The study was carried out in accordance with the recommendation of the Ethical Standard of Research Committee of Bangladesh Agricultural University, Mymensingh. The protocol and procedures employed were reviewed and approved by the Ethical Standard of Research Committee (Ref. No. BAURES/ESRC/699/2020; Dated: 28.06.2020). The authors also confirm that the ethical policies of the journal, as noted on the journal's author guidelines page, have been adhered to and the appropriate ethical review committee approval has been received. The US National Research Council's guidelines for the Care and Use of Laboratory Animals were followed.

### 2.3 Preparation of PPR virus inoculum

For experimental infection, PPR virus inoculum was prepared from a local isolate (BD/PPRV/2015/1; lineage IV) collected during a field outbreak in 2015. The virus was isolated in primary goat kidney cell culture. Briefly, a 20% tissue homogenate of lymph node was prepared from a PPR‐infected goat using sterile PBS. A monolayer of primary goat kidney cells was then infected with 100 µl of the tissue homogenate and monitored daily for the development of any cytopathic effect (CPE). The CPE was first observed at 48 hr post‐infection. The cell culture supernatant was harvested after three cycles of freeze‐thawing and cleared by centrifugation at 3,000 rpm for 15 min. The presence of PPR virus in the cell culture supernatant was confirmed by real‐time reverse transcription polymerase chain reaction (rtRT‐PCR) as described below. The virus was passaged one more time in primary goat kidney cell culture and stored in aliquots at −80°C. Before use for experimental infection of goats, the concentration of virus in the inoculum was measured by end‐point titration in primary goat kidney cell culture.

### 2.4 Experimental infection and sample collection

Goats were divided into two groups: infected (*n* = 8) and uninfected control (*n* = 4) and housed separately. Goats from the infected group received 2 ml of the PPRV inoculum containing 5.6 log_10_ TCID_50_/ml via the intranasal route. Control goats received 2 ml sterile PBS via the intranasal route. Goats from both groups were monitored twice daily for feed intake, clinical signs, body temperature and oral lesion development. Blood samples were collected at 0, 1, 2, 3, 5, 7, 10, 14, 18 and 21 days post‐infection (dpi). After collection, 2 ml of blood was transferred to a sterile vial containing ethylenediaminetetraacetic acid (EDTA) @ 1 mg/ml blood for routine haematological examination. Five millilitre of blood was also transferred to another sterile tube without EDTA. After clotting of the blood, sera were collected and centrifuged at 900 *g* for 10 min. The supernatant was transferred to fresh tubes and stored at −20°C until use.

### 2.5 Competitive enzyme‐linked immunosorbent assay (ELISA)

A competitive ELISA kit (ID Screen PPR Competition, ID‐Vet, Montpellier, France) was used to measure the amount of PPRV‐specific antibodies in sera of PPRV‐infected and healthy goats. As mentioned in the kit, a sample having a competition percentage (CP) value of ≤35 was considered as seropositive.

### 2.6 Quantification of PPR virus load in different tissues

One PPRV‐infected goat was euthanized at each time point of 5, 7, 14 and 18 dpi. Routine necropsy was performed and gross lesions were recorded. Tissues from pre‐scapular lymph node, liver, kidney and spleen were collected for quantification of PPRV RNA in tissues. To this end, a 20% tissue homogenate was prepared in sterile PBS. Total RNA was extracted with PureLink RNA Mini Kit (ThermoFisher Scientific, USA) and quantified by NanoDrop 2000 spectrophotometer (ThermoFisher Scientific, USA). For normalization and internal control, 20 ng of total RNA was used per reaction in all rtRT‐PCR. The rtRT‐PCR was performed in Applied Biosystems 7500 Fast Real Time PCR system using RevTrans QPCR One‐Step EvaGreen (ROX) Kit (Bio‐sell, Germany). The following primer pairs were used: NrF1 5′‐TGA CCA GGG AAG AAG TCA CA‐3′ and NrR1 5′‐TCG TCT TCA GGC ATG ATC TC‐3′ to amplify 120 bp product of nucleoprotein (N) gene of PPRV (Saravanan et al., 2004).

### 2.7 Haematobiochemical analysis

Routine haematological examination of whole blood samples collected at different time points post‐infection was performed by the standard method (Lamberg & Rothstein, 1978). Routine haematological parameters such as haemoglobin, erythrocyte sedimentation rate, packed cell volume, total erythrocyte count, total leucocyte count and differential leukocyte count were tested. In addition, different serum biochemical constituents such as total protein, albumin, glucose, bilirubin, blood urea nitrogen, creatine kinase, alkaline phosphatase, alanine transaminase and aspartate transaminase, inorganic phosphorus and calcium were analysed using an automated T80 Ultraviolet‐visible spectroscopy (UV/VIS) spectrophotometer (PG Instruments, UK). Furthermore, an automated electrolyte analyser GENLYTE 3000A (IVD) was used to analyse serum electrolytes (sodium, potassium and chloride ions) using a commercial kit (Electrolyte solution, Biogen, GmbH, Germany).

### 2.8 Statistical analysis

Statistical analysis was performed using the software package GraphPad Prism Version 5.0. One‐tailed non‐parametric Mann–Whitney *U* test was used to calculate the statistical differences in haematological and biochemical profiles between PPRV‐infected and healthy goats.

## 3 RESULTS

### 3.1 Host responses

**FIGURE 1:**
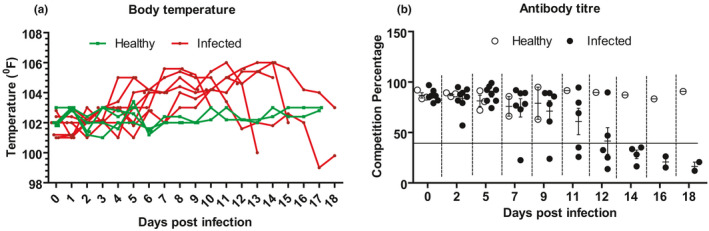
Gradual changes in the (a) body temperature and (b) antibody titre of PPRV‐infected and healthy Black Bengal goats. (b) Data indicate mean ± *SEM* of 2–8 animals

The PPRV‐infected goat showed mild depression and scanty nasal secretions starting at 4 dpi which was followed by high fever (104–105°F; Figure 1a) and watery oculonasal discharge for the next 3–5 dpi. Body temperature further increased (106°F) during 9–13 dpi with severe anorexia, dullness, dyspnoea, stomatitis, profuse orinasal and ocular discharge and diarrhoea. At 14 dpi, infected goats showed severe dyspnoea, profuse diarrhoea and orinasal inflammation with normal or sub‐normal body temperature followed by death. Goats from the control group were healthy with normal body temperature throughout the study period. In this experiment, 3 of 8 goats died and the rest of the goats were sacrificed at different time intervals as mentioned earlier. Therefore, actual mortality was not calculated. However, the morbidity was 100%. At necropsy, the infected goat showed haemorrhages and congestion in respiratory, digestive and lymphoid organs. Oral mucosa, tongue and nostrils showed erosive necrosis at 10 dpi and the severity increased over time. The trachea was congested at 7 dpi and filled with white froth. The lungs showed gradual consolidation and almost a major part of it was consolidated at advance stages. The enlarged and oedematous spleen was found in infected goats at 10 dpi, which became pale and atrophied at 14 dpi. White to greyish necrotic spot on the outer surface of the liver was found at 7 dpi. The kidneys in few infected goats were severely haemorrhagic and inflamed. On histopathology, the liver showed fatty change and single hepatocellular necrosis at an early stage which became multicellular at advance stage. The kidneys showed necrotic tubules, fusions of tubular epithelial cells and haemorrhages. The competitive ELISA of sera collected from PPRV‐infected goats showed early seroconversion stating at 7 dpi in few animals (Figure 1b). Eventually all survived goats were seroconverted by 14 dpi (Figure 1b).

### 3.2 Quantification of virus load in tissues

The virus load was determined in different tissues of PPRV‐infected goats by rtRT‐PCR. Cycle threshold (Ct) values equal to or less than 29 (Ct ≤ 29) were considered as strongly positive, 30–35 as moderate positive, 36–38 as weak positive and ≥39 as negative. Pre‐scapular lymph node (PSLN) of infected goats were strongly positive at 5 dpi (Ct 25.92) and 14 dpi (Ct 26.5), moderately positive at 7 dpi (Ct 33.71) and 18 dpi (Ct 34.2). The liver and spleen showed moderate virus loads throughout the study period except 14 dpi at which a relatively higher virus load was found (Ct values of 28.85 and 28.23 for liver and spleen, respectively). Kidneys showed a moderate virus load throughout the study period with Ct value around 30.

### 3.3 Sequential haematological changes in experimental PPRV‐infected goats

**FIGURE 2:**
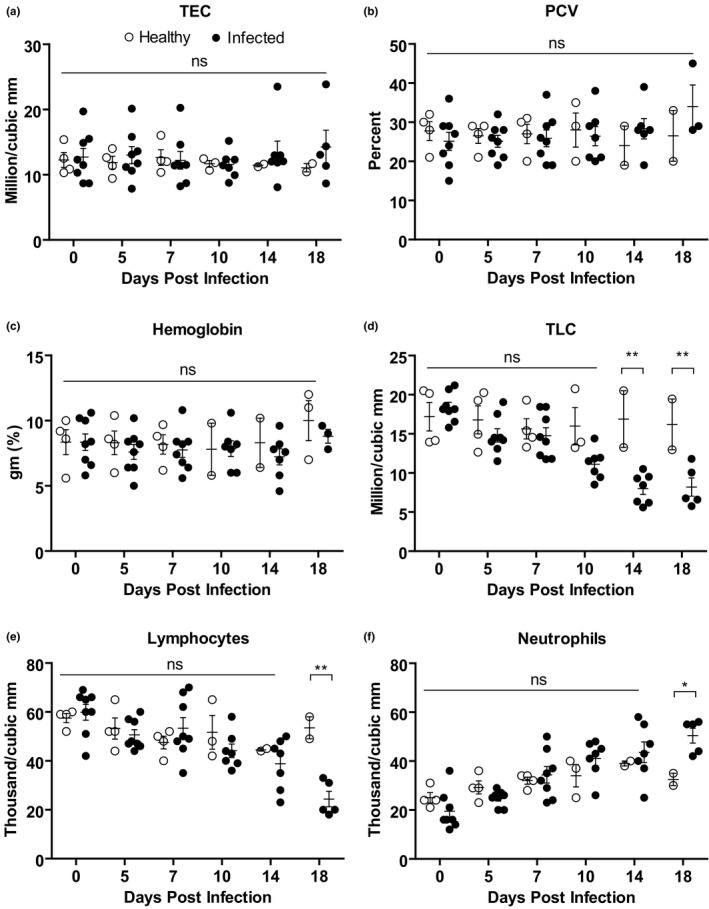
Haematological profile of PPRV‐infected Black Bengal goats. Blood samples were collected from healthy and infected goats and the (a) total erythrocyte counts, (b) packed cell volume, (c) haemoglobin level, (d) leucocyte count, (e) lymphocyte count, and (f) neutrophil count are shown. Data indicate mean ± *SEM* of 2–8 animals. One‐tailed Mann–Whitney test. *p* ≤ 0.05 indicates statistical significance

The sequential changes in haematological parameters were analysed in PPRV‐infected Black Bengal goats and compared them with that of healthy controls. Both infected and control goats showed comparable values of total erythrocyte count (TEC), packed cell volume (PCV) and haemoglobin concentration during the study period (Figure 2a–c). However, the number of total leukocyte count (TLC) decreased significantly in infected goats compared with control at 14 and 18 dpi (Figure 2d). Analysis of differential leukocyte count (DLC) revealed a significant reduction in the number of lymphocytes in infected goats at 18 dpi (Figure 2e). On the contrary, there was a slight increase in the number of neutrophils in infected goats at 18 dpi (Figure 2f). However, the number of monocytes, eosinophil and basophil remained comparable between infected and healthy goats (Figure S1). Taken together, PPRV infection showed lymphocytic leukopenia in Black Bengal goats.

### 3.4 Serum biochemical analysis of experimental PPRV‐infected goats

**FIGURE 3:**
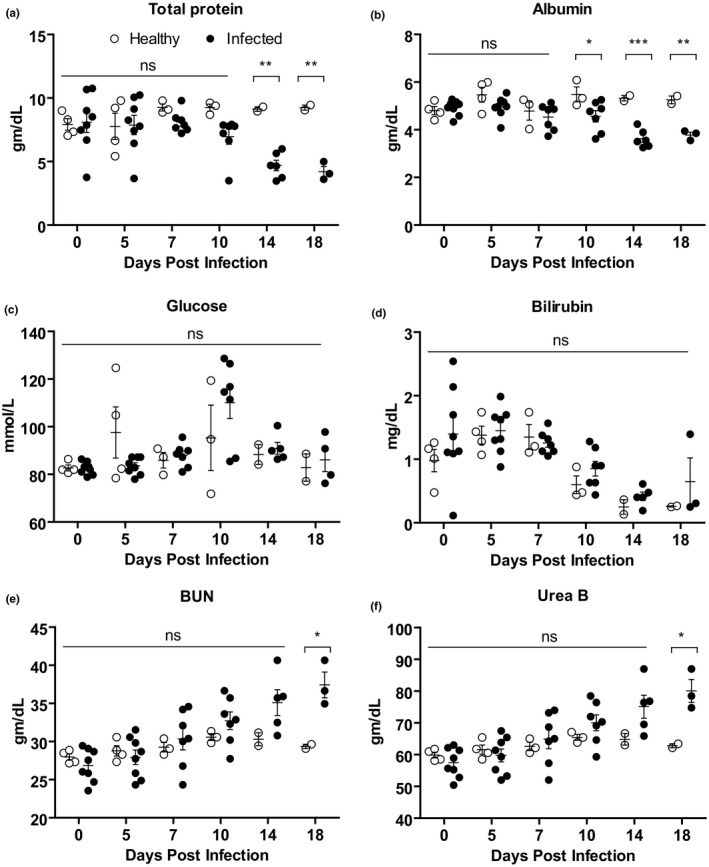
The serum biochemical profile of PPRV‐infected Black Bengal goats. Blood samples were collected from healthy and infected goats and the amount of (a) total protein, (b) albumin, (c) glucose, (d) bilirubin, (e) BUN and (g) Urea B are shown. Data indicate mean ± *SEM* of 2–8 animals. One‐tailed Mann–Whitney test. *p* ≤ 0.05 indicates statistical significance

The progressive changes in serum biochemical parameters of PPRV‐infected goats were analysed. Black Bengal goats infected with PPRV showed a significantly lower amount of total protein (hypoproteinemia) starting at 14 dpi (Figure 3a) and albumin starting at 10 dpi (Figure 3b) compared with uninfected goats. No significant differences were found in glucose (Figure 3c) and bilirubin (Figure 3d) concentration between infected and healthy goats. Analysis of blood urea nitrogen (BUN; Figure 3e) and urea B (Figure 3f) showed a gradual increase in the concentration of BUN and Urea B in sera of infected goats at 7 dpi with significant differences at 18 dpi.

**FIGURE 4:**
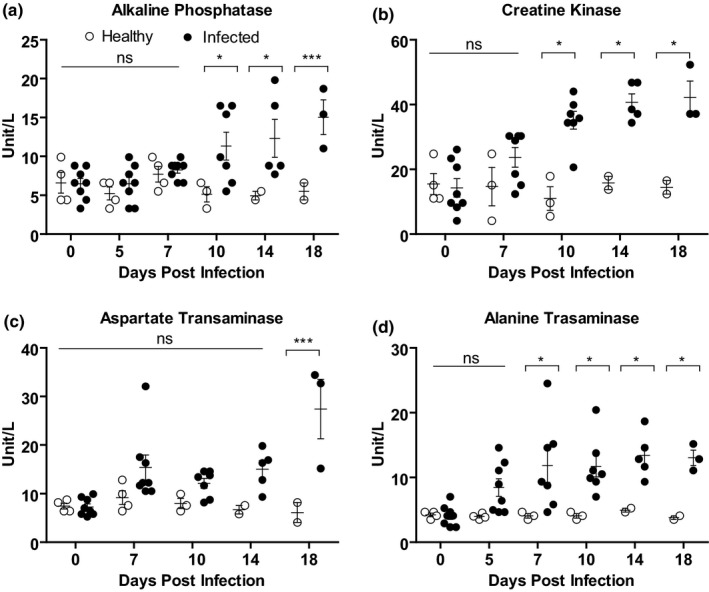
Serum enzymes level in PPRV‐infected Black Bengal goats. Blood samples were collected from healthy and infected goats and the amount of (a) alkaline phosphatase, (b) creatine kinase, (c) aspartate transaminase and (d) alanine transaminase are shown. Data indicate mean ± *SEM* of 2–8 animals. One‐tailed Mann–Whitney test. *p* ≤ 0.05 indicates statistical significance

Furthermore, four important enzymes associated with liver and kidney functions were analysed in the PPR‐infected goats. A gradual increase in the concentration of all four enzymes tested such as alkaline phosphatase (AP), creatine kinase (CK), aspartate transaminase (AST) and alanine transaminase (ALT) was found in sera of PPRV‐infected goats compared with healthy goats. The level of both AP (Figure 4a) and CK (Figure 4b) showed up‐regulation in infected goats with statistically significant differences at 10 dpi and onward. The concentration of AST increased significantly at 18 dpi in infected goats compared withhealthy goats (Figure 4c). The concentration of another serum enzyme ALT also increased significantly starting at 7 dpi in infected goats (Figure 4d). Thus, PPR affected goats showed elevated serum enzymes.

### 3.5 Analysis of serum electrolytes of experimental PPRV‐infected goats

**FIGURE 5:**
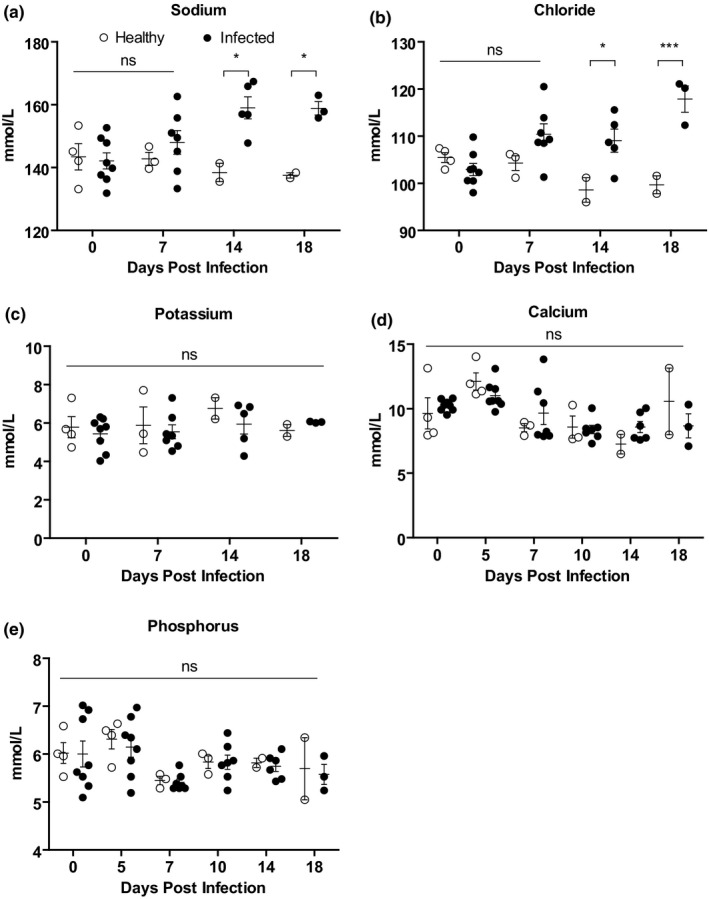
Serum electrolyte analysis of PPRV‐infected Black Bengal goats. Blood samples were collected from healthy and infected goats and the amount of (a) sodium, (b) chloride, (c) potassium, (d) calcium and (e) phosphorus are shown. Data indicate mean ± *SEM* of 2–8 animals. One‐tailed Mann–Whitney test. *p* ≤ 0.05 indicates statistical significance

The progressive changes in serum electrolytes such as sodium, potassium, chloride, calcium and phosphorus were then analysed in PPRV‐infected goats compared with healthy goats. A gradual elevation in sodium (Figure 5a) and chloride (Figure 5b) ions was found in infected goats (at 7 dpi) compared with healthy goats with significant differences at 14 and 18 dpi. However, the values of other serum electrolytes such as potassium (Figure 5c), calcium (Figure 5d) and phosphorus (Figure 5e) remained comparable between infected and healthy goats.

## 4 DISCUSSION

Experimental PPR infection induced early fever in Black Bengal goats starting at 4 dpi which became severe during 9–13 dpi with dyspnoea, cheilitis, stomatitis and gingivitis leading to reduced feed intake. The significant rise in body temperature together with respiratory distress in PPR‐infected goats were also found in several recent studies (Begum et al., 2018; Chowdhury et al., 2014; Truong et al., 2014; Ugochukwu et al., 2018). However, during the advanced stage of the disease (>14 dpi), diarrhoea was prominent in the infected goats leading to dehydration, hypothermia and death.

The majority of the study describing natural PPR outbreaks in goats showed severe anaemia attributed to massive haemorrhages in different visceral organs and disseminated intravascular clot (Begum et al., 2018; Das et al., 2015; Sahinduran et al., 2012; Sharma et al., 2012). However, we found a comparable number of total erythrocytes and haemoglobin concentrations in both infected and control goats throughout the experimental period. Such variation could be due to good management, routine deworming and good nutritional status of the animals in the present experimental study (Begum et al., 2018; Sharma et al., 2012). Few studies also reported an elevation in the amount of total erythrocyte number and haemoglobin concentration in goats died of natural PPR outbreaks which could be due to diarrhoea leading to dehydration and haemoconcentration (Islam et al., 2018; Kataria et al., 2007). Analysis of leukocyte counts showed a significant decrease in the number of total leukocytes at 14 and 18 dpi in infected goats which was marked by lymphopenia at 18 dpi similar to what has been shown in natural PPR outbreaks (Begum et al., 2018; Islam et al., 2018; Kataria et al., 2007). Such reduction in leukocyte counts could be due to initial proliferation of the virus in lymphoid organs followed by destruction of the lymphocytes as the virus has an affinity to lymphocytes (Aruni et al., 1998), as well as bone marrow suppression (Obi & Oduye, 1985). In line with this, we found significant lymphoid destruction with high virus load in lymph nodes and spleen of PPR‐infected goats. However, the number of neutrophils increased slightly in the PPR‐infected goats at 18 dpi, suggesting secondary bacterial infections (Begum et al., 2018; Islam et al., 2018; Kataria et al., 2007).

Alongside, total protein and albumin decreased after 10 dpi and urea B and blood urea nitrogen also increased at 18 dpi, suggesting liver and kidney dysfunction (Al‐Dubaib, 2009; Begum et al., 2018; Chowdhury et al., 2014; Islam et al., 2018; Kataria et al., 2007; Ugochukwu et al., 2018). Likewise, the level of alkaline phosphatase, aspartate transaminase and alanine transaminase and creatine kinase increased significantly after 7 dpi in PPR‐infected goats. These further support liver and kidney dysfunctions. Pathological investigation also revealed hepatocellular necrosis and hepatitis in the liver and tubular necrosis in the kidney starting at 7 dpi which mirrored the elevated serum enzymes and reduced protein level. Moreover, a high virus load in the liver and kidney was also found in the infected goats. A similar phenomenon has also been recorded in Black Bengal goats died of natural PPR outbreaks (Begum et al., 2018). In line with two previous studies (Das et al., 2015; Kataria et al., 2007), we also found a significant increase in the concentration of sodium and chloride ions in PPR‐infected goats at 14 dpi owing to severe diarrhoea and dehydration (Begum et al., 2018; Chowdhury et al., 2014).

In conclusion, PPR virus‐infected goats died due to (1) electrolyte imbalance, (2) immunosuppression and (3) liver and kidney dysfunctions. The balance of electrolytes in body fluid is critical for survival in diarrheic and dehydrated animals. Therefore, restoration of fluid and electrolyte balance and administration of antibiotics to prevent secondary bacterial infection should be targeted in PPR‐infected goats during the early phase of disease, as effective humoral immunity takes 2 weeks to develop. However, as sodium concentration remains either unchanged or increased in infected goats, aqua solutions have to be infused. If temperature drops at the advance stage of the disease, dextrose saline can be provided through parenteral routes. Proper intervention such as liver‐ and nephrotonic should also be made to support liver and kidney functions. Plasma transfusion can be attempted to combat dehydration and protein loss during the advance stage of the disease.

## CONFLICTS OF INTEREST

None declared.

## AUTHOR CONTRIBUTIONS

Shahana Begum: Methodology; Software; Formal analysis; Investigation; Data curation; Writing – original draft preparation. Mohammed Nooruzzaman: Software; Formal analysis; Data curation; Writing – original draft preparation; Writing – Review and Editing. Azmary Hasnat: Formal analysis; Investigation; Data curation; Writing – original draft preparation. Mohammad Rafiqul Islam: Formal analysis; Writing – original draft preparation; Writing – Review and Editing. Emdadul Haque Chowdhury: Conceptualization; Formal analysis; Investigation; Data curation; Writing – original draft preparation; Writing – Review and Editing; Supervision; Project administration; Funding acquisition. All authors have read and agreed to the published version of the manuscript.

### PEER REVIEW

The peer review history for this article is available at https://publons.com/publon/10.1002/vms3.373.

## Supporting information

Fig S1

## References

[vms3373-bib-0001] Ahmed, S. (2017). Sustainable goat farming for livelihood improvement in Bangladesh: Opportunities, constrains and potential. SAARC Agriculture Centre.

[vms3373-bib-0002] Al‐Dubaib, M. A. (2009). Peste des petitis ruminants morbillivirus infection in lambs and young goats at Qassim region, Saudi Arabia. Tropical Animal Health and Production, 41(2), 217–220. 10.1007/s11250-008-9178-6 18504645

[vms3373-bib-0003] Aruni, A. W. , Lalitha, P. S. , Mohan, A. C. , Chitravelu, P. , & Anbumani, S. P. (1998). Histopathological study of a natural outbreak of Peste des petits ruminants in goats of Tamilnadu. Small Ruminant Research, 28(3), 233–240. 10.1016/S0921-4488(97)00095-3

[vms3373-bib-0004] BBS . (2019). Bangladesh Bureau of statistics. Ministry of Planning, Government of the Peoples' Republic of Bangladesh.

[vms3373-bib-0005] Begum, S. , Nooruzzaman, M. , Parvin, M. , Mohanto, N. , Parvin, R. , Islam, M. R. , & Chowdhury, E. H. (2018). Peste des petits ruminants virus infection of Black Bengal goats showed altered haematological and serum biochemical profiles. Onderstepoort Journal of Veterinary Research, 85(1), e1–e10. 10.4102/ojvr.v85i1.1595 PMC6324080 30326714

[vms3373-bib-0006] Bhuiyan, A. R. , Chowdhury, E. H. , Kwiatek, O. , Parvin, R. , Rahman, M. M. , Islam, M. R. , & Libeau, G. (2014). Dried fluid spots for peste des petits ruminants virus load evaluation allowing for non‐invasive diagnosis and genotyping. BMC Veterinary Research, 10, 247. 10.1186/s12917-014-0247-y 25301058 PMC4203889

[vms3373-bib-0007] Chowdhury, E. H. , Bhuiyan, A. R. , Rahman, M. M. , Siddique, M. S. , & Islam, M. R. (2014). Natural peste des petits ruminants virus infection in Black Bengal goats: Virological, pathological and immunohistochemical investigation. BMC Veterinary Research, 10, 263. 10.1186/s12917-014-0263-y 25394771 PMC4233235

[vms3373-bib-0008] Das, S. , Nath, R. , Balamurugan, V. , Choudhury, R. , & Devi, M. (2015). Haemato‐biochemical analysis of goats naturally infected with Peste Des Petits ruminants. International Journal for Research in Emerging Science and Technology, 2(9), 19–24.

[vms3373-bib-0009] Haider, N. , Khan, S. U. , Islam, A. , Osmani, M. G. , Rahman, M. Z. , Epstein, J. H. , & Zeidner, N. S. (2017). Efficiency of the clinical veterinary diagnostic practices and drug choices for infectious diseases in livestock in Bangladesh. Transboundary and Emerging Diseases, 64(4), 1329–1333. 10.1111/tbed.12502 27062143

[vms3373-bib-0010] Islam, M. , Pathak, D. C. , Das, S. , Rahman, T. , Sarma, S. , & Borgohaign, R. (2018). Hematological and biochemical alterations in goats due to paste des petits ruminant's viral infection. Journal of Entomology and Zoology Studies, 6(3), 710–713.

[vms3373-bib-0011] Islam, M. R. , Shamsuddin, M. , Rahman, M. A. , Das, P. M. , & Dewan, M. L. (2001). An outbreak of peste des petits ruminants in Black Bengal Goats in Mymensngh, Bangladesh. The Bangladesh Veterinarian, 18(1), 14–19.

[vms3373-bib-0012] Kataria, A. , Kataria, N. , & Kumar Gahlot, A. (2007). Large scale outbreaks of Peste Des Petits ruminants in sheep and goats in Thar desert of India. Slovenian Veterinary Research, 44(4), 123–132.

[vms3373-bib-0013] Lamberg, S. L. , & Rothstein, R. (1978). Laboratory manual of hematology and urinalysis (3rd ed.). AVI Publishing Company.

[vms3373-bib-0014] Obi, T. U. , & Oduye, O. O. (1985). Haematological changes in natural and experimental peste des petits ruminants virus infection in goats. Revue D'elevage Et De Medecine Veterinaire Des Pays Tropicaux, 38(1), 11–15. 3837920

[vms3373-bib-0015] Rahman, M. A. , Shadmin, I. , Noor, M. , Parvin, R. , Chowdhury, E. H. , & Islam, M. R. (2011). Peste des petits ruminants virus infection of goats in Bangladesh: Pathological investigation, molecular detection and isolation of the virus. The Bangladesh Veterinarian, 28(1), 1–7.

[vms3373-bib-0016] Rahman, M. M. , Parvin, R. , Bhuiyan, A. R. , Giasuddin, M. , Chowdhury, S. M. Z. H. , Islam, M. R. , & Chowdhury, E. H. (2016). Genetic characterization of peste des petits ruminants virus circulating in Bangladesh. British Journal of Virology, 3(4), 115–122.

[vms3373-bib-0017] Sahinduran, S. , Albay, M. K. , Sezer, K. , Ozmen, O. , Mamak, N. , Haligur, M. , & Yildiz, R. (2012). Coagulation profile, haematological and biochemical changes in kids naturally infected with peste des petits ruminants. Tropical Animal Health and Production, 44(3), 453–457. 10.1007/s11250-011-9917-y 21732067

[vms3373-bib-0018] Saliki, J. T. (1988). Peste des petits ruminants (6th ed.). US Animal Health Association, Committee on Foreign Animals Diseases.

[vms3373-bib-0019] Saravanan, P. , Singh, R. P. , Vinayagamurthy, B. , Dhar, P. , Bp, S. , Dhanavelu, M. , & Bandyopadhyay, S. (2004). Development of a N gene‐based PCR‐ELISA for detection of Peste‐des‐petits‐ruminants virus in clinical samples. Acta Virologica, 48, 249–255. 15745048

[vms3373-bib-0020] Sharma, C. , Mehta, H. , Prakash, M. , & Shukla, P. (2012). Studies on clinico‐haemato‐biochemical changes in peste des petits ruminants in goats. Veterinary Practitioner, 13(2), 322–325.

[vms3373-bib-0021] Siddiky, M. N. A. (2013). Economic impact of transboundary animal diseases in SAARC countries. SAARC Agriculture Centre.

[vms3373-bib-0022] Truong, T. , Boshra, H. , Embury‐Hyatt, C. , Nfon, C. , Gerdts, V. , Tikoo, S. , & Babiuk, S. (2014). Peste des petits ruminants virus tissue tropism and pathogenesis in sheep and goats following experimental infection. PLoS One, 9(1), e87145. 10.1371/journal.pone.0087145 24498032 PMC3907444

[vms3373-bib-0023] Ugochukwu, I. C. I. , Ugochukwu, E. I. , & Chukwu, C. C. (2018). Haematological parameters and serum biochemical assay of West African Dwarf goats infected with peste des petits ruminants virus in Nsukka, Enugu State. Comparative Clinical Pathology, 27(1), 13–19. 10.1007/s00580-017-2545-9

[vms3373-bib-0024] Yousuf, M. A. , Giasuddin, M. , Islam, S. S. , & Islam, M. R. (2015). Management of an outbreak of peste des petits ruminants with antibiotic combined hyperimmune serum therapy. Asian Journal of Medical and Biological Research, 1(2), 230–234. 10.3329/ajmbr.v1i2.25616

